# Urinary α 1-microglobulin and β 2-microglobulin as markers of early kidney injury in HIV-positive male patients on tenofovir-based antiretroviral therapy

**DOI:** 10.1371/journal.pone.0303442

**Published:** 2024-06-17

**Authors:** Xiao Li Yu, Wen Sun, Li Liu, Ke Hong, Hui Song

**Affiliations:** 1 The Fourth Department of Infectious Diseases, Wuhan Jinyintan Hospital, Tongji Medical College of Huazhong University of Science and Technology, Wuhan, China; 2 Hubei Clinical Research Center for Infectious Diseases, Wuhan, China; 3 Wuhan Research Center for Communicable Disease Diagnosis and Treatment, Chinese Academy of Medical Sciences, Wuhan, China; 4 Joint Laboratory of Infectious Diseases and Health, Wuhan Institute of Virology and Wuhan Jinyintan Hospital, Chinese Academy of Sciences, Wuhan, China; University of Ghana, GHANA

## Abstract

**Background:**

A retrospective study was conducted to explore the urinary expression of α 1-microglobulin (α1MG) and β2-microglobulin (β2MG) in patients with human immunodeficiency virus (HIV) infection, aiming to evaluate their predictive capability for renal injury.

**Method:**

One hundred and five male HIV-infected patients treated with Tenofovir (TDF) regimen (TDF+3TC or the third drug TDF/FTC+) were selected between March 1, 2021, and March 1, 2022, in Wuhan Jinyintan Hospital. Three months after TDF treatment, the renal function injury was evaluated with the standard creatinine clearance rate. The urinary levels of α1MG and β2MG were compared between the initiation of TDF treatment and three months thereafter. Spearman correlation was utilized to analyze the correlation between the urinary expression of α1MG and β2MG and renal injury in HIV patients. The logistic regression was used to analyze the predictive value of urinary α1MG and β 2-microglobulin expression in renal injury.

**Results:**

Up to the first follow-up, 29 (27.6%) cases of the 105 male HIV patients had varying degrees of renal function injury, including 14 (13.3%) mild injury, 9 (8.6%) moderate injury, and 6 (5.7%) severe injury cases. Patients with severe renal injury had the highest levels of urinary α1MG and β2MG expression while those with mild injury demonstrated higher levels compared to the non-injury group (*P* < 0.05). Spearman correlation analysis indicated that urinary α1MG and β2MG were positively correlated with renal impairment in HIV patients (Rho = -0.568, and -0.732; *P* < 0.001). The ROC curve analysis demonstrated that the area under the curve (AUC) for urine α1MG and β2MG in predicting kidney damage among HIV patients were 0.928, 0.916, and 0.889, respectively. The sensitivity values were 96.55%, 82.76%, and 89.66% while the specificity values were 84.07%, 94.51%, and 89.29% for urine α1MG and β2MG, respectively.

**Conclusion:**

The expression level of urinary α1MG and β2MG in HIV patients was significantly higher compared to normal people. Detection of these two indexes can enable early determination of renal injury and its severity in HIV patients.

## Introduction

Tenofovir (TDF) is a novel nucleotide reverse transcriptase inhibitor utilized in the treatment of viral infectious diseases [1]. In 2012, TDF was clinically employed as a first-line treatment and provided free of charge for HIV infection in China. Due to its potent antiviral effect and low drug resistance, TDF has been widely adopted in clinical, establishing itself as a first-line medication for treating HIV infection [2]. The incidence of TDF-induced nephrotoxicity is relatively lower among individuals of other racial backgrounds [3,4]. However, there are several reports that TDF nephrotoxicity is relatively common among Asians attributed to their lower body weights [5–7]; and it is strongly recommended to monitor renal function prior to initiating TDF in Asians [8].

The toxicity of TDF is well-known for its involvement in the proximal renal tubules. Meanwhile, the estimated glomerular filtration rate (eGFR) has traditionally served as a means to assess the glomerular function in individuals, playing a crucial role as a functional index in diagnosing and staging chronic kidney disease (CKD) [9,10]. Biomarkers indicating tubular pathology have been demonstrated to be more sensitive compared to eGFR detection as tubular dysfunction often precedes the decline in eGFR [11–15]. A number of biomarkers have been reported in early diagnosis of TDF-induced kidney tubular dysfunction (KTD), including α1-microglobulinuria (α1MG), β2-microglobulinuria (β2MG), kidney injury marker-1 (KIM-1), N-acetyl- β-D-glucosaminidase (NAG), fractional excretion of phosphate (EFIP), and fractional excretion of uric acid (FEUA) [14,16–18]. In practice, monitoring multiple biomarkers is complex and costly with variations evident in each study. Additionally, there remains a limited of studies investigating biomarkers of KTD among HIV-infected TDF users in Asia to date. According to the clinic experience of our hospital, most of the HIV patients were male and performed TDF treatment recent years.

The aim of this study was that we tried to monitor and evaluate the values of biomarkers (α1MG and β2MG) in identifying TDF-induced KTD among the male patients with HIV-1 infection at a Chinese hospital.

## Materials and methods

### Study design and population

This study included 105 male patients diagnosed with HIV infection at Wuhan Jinyintan Hospital. These patients received treatment with the TDF regimen (either TDF+3TC or the third drug TDF/FTC^+^) between March 1, 2021 and March 1, 2022. All patients underwent urine sample collection prior to initiating TDF treatment with at least one additional sampling conducted after receiving the TDF regimen for a duration of three months. A medical ethics committee at our hospital has approved this study. Informed consent was obtained from all study participants, and all clinical investigations adhered to the principles outlined in the 1995 Declaration of Helsinki.

Inclusion criteria were as follows: 1). age ≥ 21 years old, and male; 2). confirmation of positive HIV antibody via Western Blot testing; 3) absence of prior antiretroviral therapy (ART) history; 4). adherence to a 3-month follow-up period during which the patients did not alter their antiviral regimen and demonstrated good treatment compliance; 5). enrollment with an estimated glomerular filtration rate (eGFR) ≥ 90mL/min (calculated using CKD-EPI [19]); 6). provision of informed consent for antiviral treatment accompanied by complete baseline and follow-up information.

Exclusion criteria: 1) those with acute or chronic kidney diseases; 2) those with hepatitis B or C co-infection; 3) those with positive serum pregnancy test or planned pregnancy; 4) those who had allergic reactions or other contraindications to any component of therapeutic drugs; 5) those who dropped out of this study, or were participating in other similar researchers; 6) those with tumors and using immunosuppressive or chemoradiotherapy drugs; 7) HIV-1-active drugs had been used to treat non-HIV-1 viral infections, such as hepatitis B; 8) unconscious or complicated with mental illness.

### Methods

All HIV patients were confirmed by Western blot test (WB). The WB was performed on sera with two reactive tests as per the instruction previously described [20]. A positive result for HIV infection was established if at least two of the bands representing gp160, gp120, gp41, and gag p24 were detected. A fasting venous blood sample of 3ml was collected from each patient who had TDF treatment for 3 months. The serum was collected after 3000 rpm centrifugation for 10 minutes. The serum creatinine (Scr) level was measured using the COBAS INTEGRA 800 automatic biochemical analyzer (Roche). The standard creatinine clearance rate was estimated by the equation: {[140 –Age (years)] x Body mass (kg)}/0.818 x Serum creatinine (μ mol/L). Patients with a creatinine clearance rate ≥ 80ml/min showed no renal injury while those with rates of 50–79 ml/min exhibited mild injury. Patients with rates of 20–49 ml/min presented with moderate injury and those with rates < 20 ml/min experienced severe injury [21].

Urine samples were collected after the mixing of 24-hour urine collections. These samples underwent centrifugation at 3,000 rpm for 10 minutes to separate cellular debris and the resulting supernatants were stored at -80°C. Urinary α1-MG and β2-MG levels were measured in thawed supernatants using commercial enzyme-linked immunosorbent assay (ELISA) kits (Cusabio Technology LLC, China).

### Observation index

Three months after treatment, the levels of urinary α1MG and β2MG were compared. Patients were divided into four groups based on the degree of renal function injury (no injury, mild injury, moderate injury, and severe injury). The levels of urinary α1MG and β2MG in these four groups were then compared.

### Statistical analysis

For data analysis, SPSS22.0 (IBM, New York) was utilized. The numeric data were expressed by mean ± standard deviation ($\bar{x}$±s). The counting data was expressed by [n (%)]. The group comparisons were conducted using a Student *t*-test (normal distribution) or Mann-Whitney U test (nonnormal distribution). The correlation analysis of α1-MG/β2-MG with creatinine clearance was carried out by Spearman correlation test. Logistic regression analysis was performed to assess the risk of renal injury. The receiver operating curve (ROC) was adopted to analyze the predictive value of urinary α1-MG and β2-MG on HIV kidney injury. A *P* < 0.05 was considered statistically significant.

## Results

### Characteristics of the studied subjects

The study included a total of 105 male subjects with ages ranging from 22 to 61 years and an average age of 40.94 ± 6.94 years. The body weight ranged from 45 to 88 kg with an average of 67.91 ± 8.17 kg. All patient information regarding the patients was provided in **S1 Dataset** and summarized in **Table 1.**

**Table 1:** The information of the studied patients.

	Studied patients
Gender	
Male	105
Ages (years)	40.94 ± 6.94 (19–81)
Body weight (kg)	67.91 ± 8.17
Education	
Junior school	20
High or Technical second	32
College or more	53
Hypertension	20
Hyperlipidemia	10
Diabetes	14

### The renal function indexes between baseline stage and first follow-up in patients with HIV

After 3 months of TDF treatment, the expression levels of urinary α1MG and β2MG in the patients were 4.39 ± 3.05 mg/L and 2.42 ± 0.93 mg/L, respectively. These levels were significantly higher than those observed at treatment initiation (1.36 ± 0.39 mg/L and 1.44 ± 0.44 mg/L, respectively) (P<0.05) as illustrated in **Table 2**.

**Table 2:** Comparison of the urinary α1MG and β2MG levels in HIV patients with TFD initiation and 3 months after TFD treatment.

Group (n = 105)	Urinary α1-MG (mg/L)	Urinary β2-MG (mg/L)
TFD initiation	± 0.39	1.44 ± 0.44
3 months after TFD treatment	4.39 ± 3.05	2.42 ± 0.93
*Z*	- 12.44 ^a^	- 8.83 ^a^
*P*	**<0.01**	**<0.01**

^a^ Mann-Whitney U test; MG: Macroglobulin; Bold indicates the statistically significant.

### The expression levels of urinary α1-microglobulin and β2-microglobulin in HIV patients with different degrees of renal function injury

None of the patients with comorbidities such as diabetes mellitus, hypertension, and hyperlipidemia developed different degrees of renal function injury. Among the 105 HIV-infected patients receiving TDF treatments, 29 cases (27%) exhibited varying degrees of renal function injury, including mild, moderate, and severe injury as outlined in **Table 3**. The urinary α1MG and β2MG expression levels of the four groups of HIV patients were significantly different (*P* < 0.05). Compared to the non-injury patients, the severe injury group exhibited the highest expression levels of urinary α1MG (15.14 ± 5.41 mg/L) and β2MG (4.55 ± 0.49 mg/L), respectively. Additionally, the urinary α1MG and β2MG levels in the mild injury group were much higher (4.24 ± 0.21 mg/L and 2.81 ± 0.42 mg/L, respectively), which were also higher compared to the non-injury group (refer to **Table 3)**.

**Table 3:** The expression levels of urinary α1MG and β2MG in HIV-positive patients after TFD treatment.

Grading of renal function injury	N (%)	Urinary α1MG (mg/L)	*P* value^a^	Urinary β2MG (mg/L)	*P* value^a^
Non-invasive group	76 (72.4)	3.44 ± 0.68	-	2.05 ± 0.57	-
Mild injury group	14 (13.3)	4.24 ± 0.21	**<0.01**	2.81 ± 0.42	**< 0.01**
Moderate injury group	9 (8.6)	5.50 ± 1.32	**< 0.01**	3.76 ± 0.98	**< 0.01**
Severe injury group	6 (5.7)	15.14 ±5.41	**< 0.01**	4.55 ± 0.49	**< 0.01**

^a^ Student *t* test; MG: Macroglobulin; Bold indicates the statistically significant.

### Correlation between urinary α1-microglobulin, β 2-microglobulin and grading of renal function injury in patients with HIV

**Fig 1:**
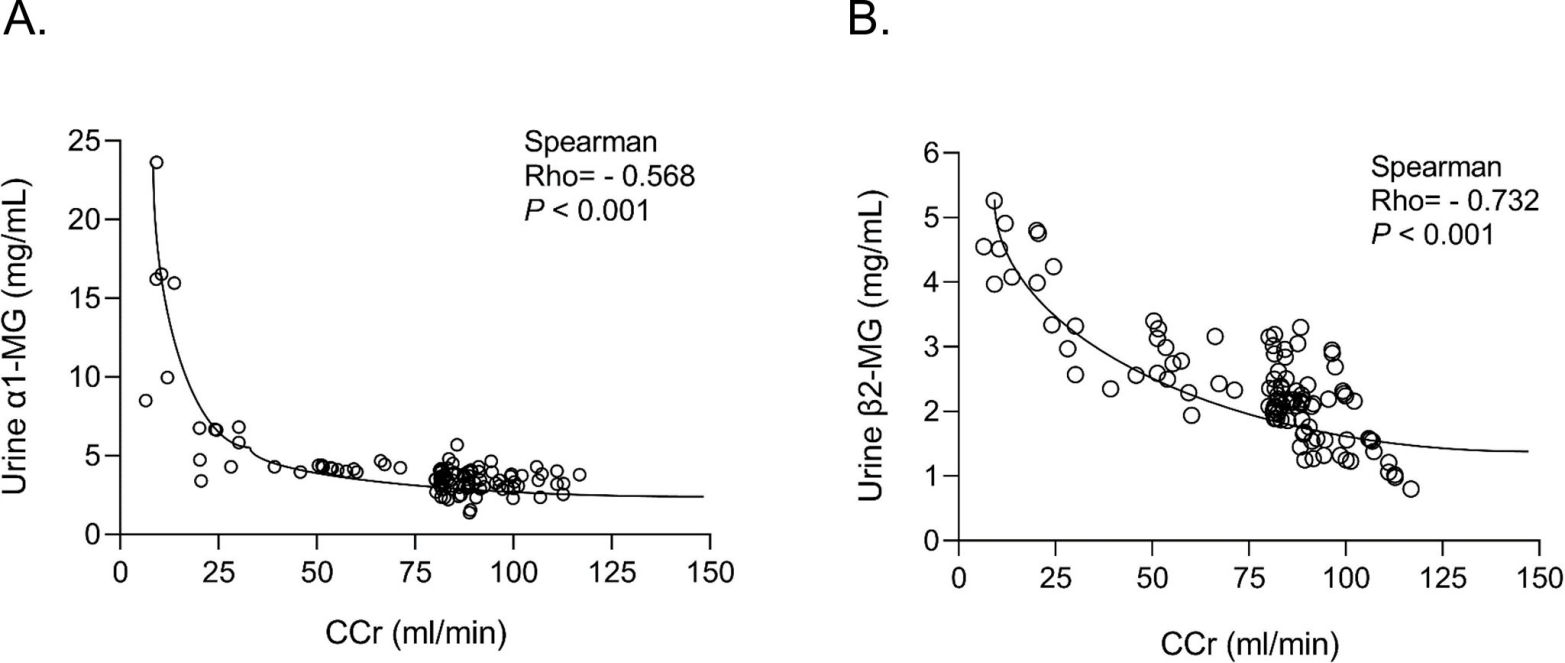
Correlation between levels of urinary α1-MG, β2-MG and creatinine clearance (or eGFR) of HIV patients with TDF treatment. (**A**). level of urine α1-MG versus CCr; (**B**): Level of urine β2-MG versus CCr. MG: Macroglobulin; CCr: Creatinine clearance rate; Rho: Spearman correlation coefficient.

To explore the relationship between renal function and levels of α1MG and β2MG, we conducted a Spearman correlation analysis on the data. The results indicated that urinary α1MG and β2MG were positively correlated with the renal function impairment in HIV patients treated with TDF (Rho = -0.568, and -0.732, respectively; *P* < 0.001) as shown in **Fig 1**.

### Predictive value of urinary α 1-microglobulin and β 2-microglobulin on renal injury in HIV patients with TDF treatments

A univariate logistic regression analysis was performed to predict the risk of renal injury using urinary α1MG and β2MG. The analysis revealed that both urinary α1MG and β2MG were capable of predicting the risk of renal injury in HIV-infected patients receiving the TDF regimen (OR = 13.75; OR = 15.64, respectively) (refer to **Table 4**). Furthermore, in multiple logistic analyses, both urinary α1MG and β2MG levels were significantly associated with renal injury (OR = 19.41–21.56) (**Table 4**). Obviously, both urinary α1MG and β2MG were identified as risk factors for renal injury.

**Table 4:** Association between urinary α1MG and β2MG levels and risk of renal injury in logistic regression analysis.

Variables	Univariate analysis			Multivariate analysis		
	OR	95% CI	*P* value	OR*	95% CI	*P* value
**α1-MG**	13.75	3.50–53.94	<0.001	19.44	3.00–125.84	0.002
**β2-MG**	15.64	4.83–50.72	<0.001	21.56	3.31–140.55	0.001

MG: Macroglobulin; OR: Odd ratio; CI: Confidence interval

*: Odd ratios were adjusted by age and BMI. A *p* value less than 0.05 is considered statistically significant.

**Fig 2:**
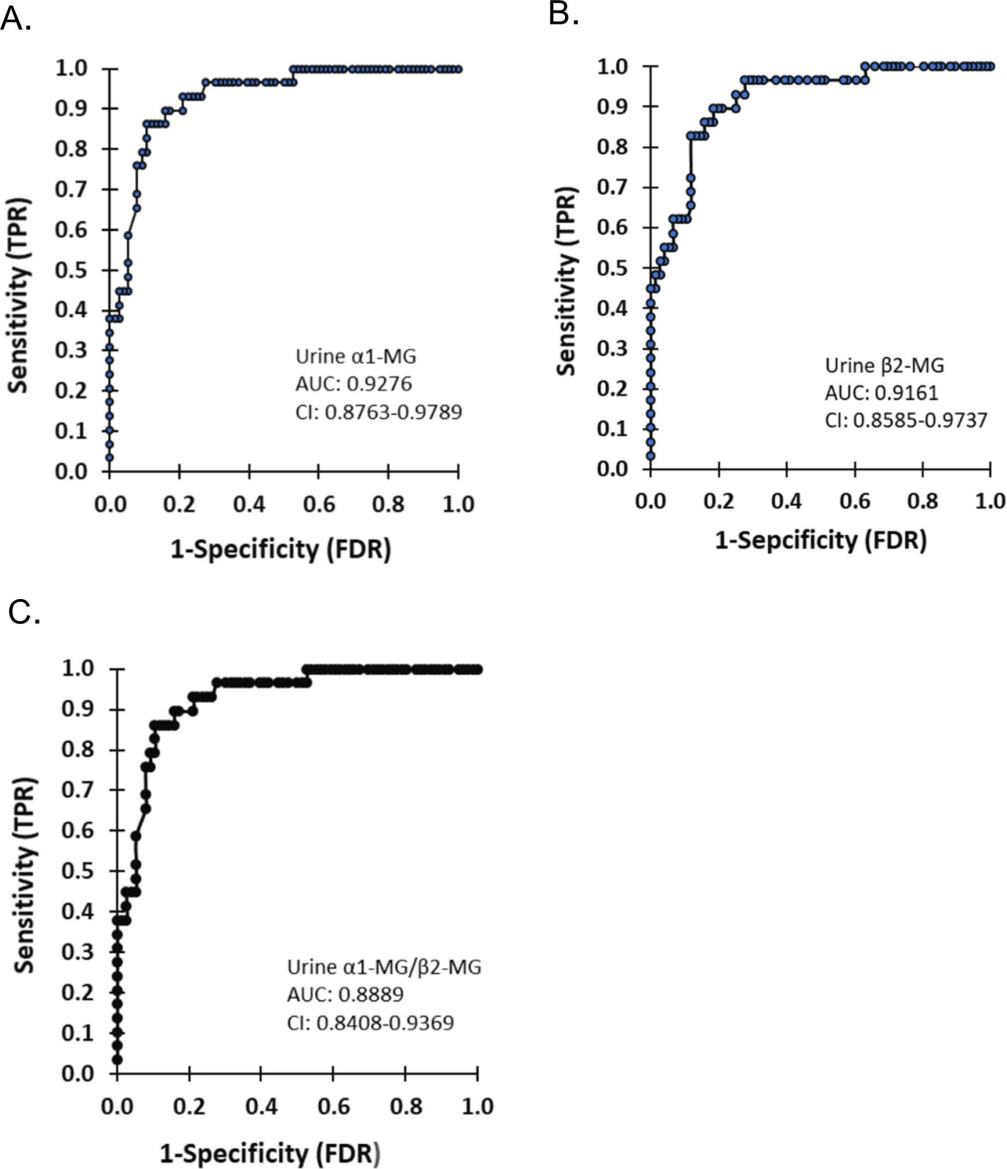
ROC curve of urinary α 1-microglobulin and β 2-microglobulin for predicting renal injury in HIV patients with TDF treatment. (**A**): Urine α1-microglobulin; (**B**): β2-microglobulin; (**C**): Combined diagnosis. ROC: Receiver operating characteristic; TPR: True positive rate; FPR: False positive rate.

The results of the ROC curve study indicated that the area under the curve (AUC) for urinary α1MG or/and β2MG in predicting renal injury among HIV-infected patients receiving TDF treatments were 0.928, 0.916, and 0.889, respectively. The corresponding sensitivity values were 96.55%, 82.76%, and 89.66%, while the specificity values were 84.07%, 94.51% and 89.29%, respectively. Further details can be found in **Fig 2** and **Table 5**.

**Table 5:** Diagnostic efficacy of detection of urinary α1MG and β2MG in renal injury in HIV patients with TDF treatment.

Variable	AUC	Sensitivity (%)	Specificity (%)	Yoden index	95%CI
Urinary α 1-MG	0.928	96.55	84.07	0.81	0.974–0.979
Urinary β 2-MG	0.916	82.76	94.51	0.77	0.859–0.974
Joint diagnosis	0.889	89.66	89.29	0.79	0.841–0.957

AUC: The area under the curve; CI: Confidence interval; MG: Micorglobulin.

## Discussion

Tenofovir (TDF) is a nucleotide reverse transcriptase inhibitor that has been introduced to treat HIV infection in China since 2013. Its once-daily dosing regimen improves patient compliance with long-term medication offering a strong antiviral effect with a low resistance rate. Consequently, it merged as a first-line medication for managing both HIV and HBV infection in China [22,23]. Overall, TDF treatments have demonstrated good safety profiles with a low incidence of renal damage [24]. However, as the treatment population increases and treatment duration extends, renal damages such as tubular necrosis, acute renal failure, and Fanconi Syndrome have merged as common clinical problems among HIV-positive patients receiving TDF-based antiretroviral therapy [6,25]. The reported incidence of TDF-related renal damage falls within a range of 4% to 7%. Specifically, the incidence of moderate renal damage is approximately 5%, while severe renal damage is reported to occur in approximately 1.7% of cases [26]. In the current study, the incidences of overall mild, moderate, and severe TDF-related renal damage of the male patients were found to be 13.3%, 8.6%, and 5.7%, respectively. These rates are relatively higher than those reported in previous studies. Interestingly, these findings are consistent with the previous studies conducted in Asian populations [5,7]. While various factors such as old age, low body weight, comorbidities, hepatitis infections, nephrotoxic drugs, and preexisting renal damage are known risk factors for TDF-inducted KTD. Our findings suggest that low body weight may be a major risk factor among the male patients included in our study. Therefore, our findings demonstrate that early detection of tubular toxicity induced by TDF-based antiretroviral therapy is crucial and essential for ensuring patient safety and guiding TDF treatment strategies in Asian patients with HIV, especially for the Asian male HIV cases.

We observed significant changes in urinary α1MG and β2MG concentrations among the male HIV-positive patients during the initiation and the first three months of TDF use. These changes are indicative of proximal tubular dysfunctions associated with TDF therapy. Male participants who received TDF-based therapy had significantly higher levels of urine α1MG and β2MG proteins compared to the general population. Moreover, the severe injury group exhibited the highest expression levels of urinary α1MG and β2MG while the mild injury group also demonstrated elevated levels compared to the non-injury group. Our results showed that urinary α1MG and β2MG were positively correlated with renal function impairment in HIV-positive male patients on TDF-based antiretroviral therapy (*z* = - 12.4, and -8.84, *P* < 0.01). These data indicated that the increasing levels of urinary α1MG and β2MG proteins were associated with the aggravation of renal injuries, thus reflecting the male HIV patient’s glomerular and tubular damages to a certain extent in the early stage of kidney pathologic changes as TDF therapy.

Both α1MG and β2MG are low molecular weight proteins. α1MG is a glycoprotein reabsorbed and metabolized in the proximal convoluted tubule of the kidney [27]. Most importantly, the detection of α1MG level is not affected by factors such as the pH value of urine, laboratory temperature, malignant tumors, and others [28]. β2MG is produced by lymph, platelets and other nucleated cells in the body [29]. It undergoes degradation by renal tubular endothelial cells into amino acids [30]. Both α1MG and β2MG are freely filtered by the glomerulus and subsequently reabsorbed completely in the proximal tubule. This characteristic renders these proteins highly sensitive and specific laboratory indices for different injuries of proximal renal tubule function [30]. The present study indeed demonstrated that both urine α1MG and β2MG were important markers of KTD and could be used for early diagnosis of TDF-induced KTD in the male HIV patients.

The early detection and dynamic monitoring of renal injuries in HIV patients, especially in the context of TDF use, would be of great significance [31]. Comprehensive evaluation of disease progression often improves the accuracy of early diagnosis of kidney injury in HIV patients through a combination of multiple indicators [32]. The combined detection can significantly increase the sensitivity of early diagnosis and holds significant value in formulating prevention and treatment programs [33]. The early reversibility and concealment of renal injury in HIV patients treated with TDFs is not conducive to early clinical diagnosis. Our results showed that the AUCs for urinary α1MG and β2MG detection alone as well as their combination in predicting kidney damage in TDF treated male HIV patients were 0.928, 0.916, and 0.889, respectively. The corresponding sensitivities were 96.55%, 82.76% and 89.66% while the specificities were 84.07%, 94.51%, and 89.29%, respectively. It indicated that the urinary α1MG and β2MG levels might accurately and objectively predict the development of kidney damage in male HIV patients at an early stage during TDF therapy. However, we did not find that the combined detection of urinary α1MG and β2MG could increase the AUGs, sensitivity, and specificity of the diagnosis. It might be due to small clinical samples in the detections.

The strength of this study lies in its retrospective design, which allowed for the examination of a homogeneous population selected according to predefined criteria. Additionally, all male patients were prescribed the same antiretroviral regimen (TDF + 3TC/or FTC), enabling a proper evaluation of the diagnostic accuracy of α1MG and β2MG without the confounding influence of concomitant use of other antiretroviral drugs on TDF-induced CKD. However, several limitations were noted in this study. Firstly, the sample size was small and mostly consisted of male subjects, which may introduce bias, and could not apply our findings to female cases. Secondly, this study was conducted at a single medical center, potentially leading to important variations between the study subjects and the general HIV-infected population.

## Conclusions

In conclusion, the levels of urinary α1MG and β2MG may increase in the early stages of renal injury among male HIV patients receiving TDF-based therapy in China. Detection of urinary α1M and β2M of male HIV patients can enhance the early diagnosis rate of renal injury and provide a laboratory basis for grassroots doctors in the treatment and monitoring of TDF-based therapy.

## Supporting information

S1 Dataset (XLSX)
